# Effects of Dietary Fat and Saturated Fat Content on Liver Fat and Markers of Oxidative Stress in Overweight/Obese Men and Women under Weight-Stable Conditions

**DOI:** 10.3390/nu6114678

**Published:** 2014-10-28

**Authors:** Anna Marina, Anize Delfino von Frankenberg, Seda Suvag, Holly S. Callahan, Mario Kratz, Todd L. Richards, Kristina M. Utzschneider

**Affiliations:** 1 Division of Metabolism, Endocrinology and Nutrition, Department of Medicine, University of Washington, 1959 NE Pacific Street, Seattle, WA 98195, USA; E-Mails: annam45@hotmail.com (A.M.); anize.frankenberg@gmail.com (A.D.F.); sedasuv@yahoo.com (S.S.); mkratz@fhcrc.org (M.K.); 2 School of Medicine, University of Washington, 1959 NE Pacific Street, Seattle, WA 98195, USA; E-Mail: hcal@uw.edu; 3 Fred Hutchinson Cancer Research Center, Division of Public Health Sciences, 1100 Fairview Ave N, Seattle, WA 98109, USA; 4 Department of Epidemiology, University of Washington, 1959 NE Pacific Street, Seattle, WA 98195, USA; 5 Department of Radiology, University of Washington, 1959 NE Pacific Street, Seattle, WA 98195, USA; E-Mail: toddr@u.washington.edu; 6 VA Puget Sound Health Care System, Endocrinology, 1660 S Columbian Way (151), Seattle, WA 98108, USA

**Keywords:** non-alcoholic fatty liver, dietary fat, saturated fat, oxidative stress

## Abstract

Dietary fat and oxidative stress are hypothesized to contribute to non-alcoholic fatty liver disease and progression to steatohepatitis. To determine the effects of dietary fat content on hepatic triglyceride, body fat distribution and markers of inflammation and oxidative stress, overweight/obese subjects with normal glucose tolerance consumed a control diet (CONT: 35% fat/12% saturated fat/47% carbohydrate) for ten days, followed by four weeks on a low fat (LFD (*n* = 10): 20% fat/8% saturated fat/62% carbohydrate) or high fat diet (HFD (*n* = 10): 55% fat/25% saturated fat/27% carbohydrate). Hepatic triglyceride content was quantified by MRS and abdominal fat distribution by MRI. Fasting biomarkers of inflammation (plasma hsCRP, IL-6, IL-12, TNFα, IFN-γ) and oxidative stress (urinary F2-α isoprostanes) were measured. Body weight remained stable. Compared to the CONT, hepatic triglyceride decreased on the LFD (mean (95% CI): change −2.13% (−3.74%, −0.52%)), but did not change on the HFD and there was no significant difference between the LFD and HFD. Intra-abdominal fat did not change significantly on either diet, but subcutaneous abdominal fat increased on the HFD. There were no significant changes in fasting metabolic markers, inflammatory markers and urinary F2-α isoprostanes. We conclude that in otherwise healthy overweight/obese adults under weight-neutral conditions, a diet low in fat and saturated fat has modest effects to decrease liver fat and may be beneficial. On the other hand, a diet very high in fat and saturated fat had no effect on hepatic triglyceride or markers of metabolism, inflammation and oxidative stress.

## 1. Introduction

Nonalcoholic fatty liver disease (NAFLD) is a condition in which fat accumulates in the liver in the absence of significant alcohol intake. A subset of individuals with NAFLD develop inflammation, hepatocyte injury and fibrosis (non-alcoholic steatohepatitis or NASH) which can progress to cirrhosis [1]. Combining this effect with the obesity epidemic, NASH is now projected to be the leading cause for liver transplant by 2020 [2]. Oxidative stress and inflammation are thought to be key factors in this progression [3].

Dietary fat may be an important modifiable factor in the development of NAFLD and NASH. Multiple animal models have demonstrated that high fat/saturated fat diets (HFD) increase liver fat and promote hepatocyte injury [4,5], but few well-controlled, isocaloric dietary intervention studies in humans have been performed. Two studies found that two to three weeks on a low fat/low saturated fat diet (LFD) decreased liver fat compared to a HFD [6,7], while we demonstrated in older non-diabetic adults a modest effect of four weeks on a LFD to decrease liver fat, but no effect of a HFD [8]. Based on this last study, the ability of high dietary fat intake to induce fatty liver in humans is questionable.

We hypothesized that dietary fat plays a causal role in the development of NAFLD. Thus, an isocaloric HFD should increase and a LFD should decrease hepatic triglyceride content and associated oxidative stress and inflammation compared to a control diet containing moderate amounts of fat (CONT).

## 2. Subjects and Methods

### 2.1. Study Design

This study was a prospective, random order, controlled dietary feeding study. To standardize baseline diet composition, all subjects completed a 10-day CONT diet prior to the intervention diet. The CONT was based on a standard American macronutrient composition and was similar to the average macronutrient composition reported by study subjects. Thus, 10 days was felt to be adequate time to achieve standardization. The CONT diet was then followed by four weeks on either a LFD or HFD. Seven participants completed both intervention diets, with the CONT diet being repeated before each intervention diet and a 6-week washout period separating the two diet periods. During the washout period study subjects ate their usual diet and received no food from the study.

### 2.2. Subjects

Participants were enrolled if they were 18–55 years old, with normal fasting (<100 mg/dL) and 2 h glucose (<140 mg/dL) on an oral glucose tolerance test, and BMI > 27 kg/m^2^. Subjects had to be healthy, nonsmokers, with normal liver and renal function, and drink fewer than 2 alcoholic drinks/day by self-report. Exclusion criteria included food allergies/intolerances, contraindications to MRI, and medications affecting inflammation, insulin sensitivity, or liver fat. All subjects gave written informed consent. This study was approved by the Institutional Review Boards of the Veterans Affairs Puget Sound Health Care System and the University of Washington and was in accordance with ethical standards on human experimentation.

### 2.3. Dietary Intervention

Menus were designed by a research nutritionist using ProNutra (VioCare, Inc., Princeton, NJ, USA): CONT: 35% energy from fat/12% saturated fat; LFD: 20% fat/8% saturated fat; HFD: 55% fat/25% saturated fat. Major sources of fats in all three diets included butter and high oleic safflower oil. Vegetable content was matched. Because fructose was limited on the HFD due to the low carbohydrate content, fructose was limited in all diets to <30 g/day based on a 2000 kcal/day diet (Table 1). Menus are provided in supplemental Tables S1–S3.

**Table 1:** Diet composition: baseline diet as estimated by 3-day food record and study diets consumed.

Title	Baseline ^a^	CONT LFD	LFD	CONT HFD	HFD
Daily energy (kcal)	2326 ± 334	3284 ± 125	3321 ± 150	3140 ± 120	3208 ± 92
Fat (% of total energy)	38.6 ± 2.1	35.8 ± 0.6	20.2 ± 0.003 ^1^	35.2 ± 0.02	54.8 ± 0.05 ^2,3^
Saturated fat (% of total energy)	13.2 ± 0.9	11.9 ± 0.4	7.7 ± 0.01 ^1^	11.6 ± 0.02	23.7 ± 0.05 ^2,3^
MUFA (% of total energy)	13.7 ± 1.0	16.7 ± 0.1	7.7 ± 0.01 ^1^	16.6 ± 0.04	22.2 ± 0.06 ^2,3^
PUFA (% of total energy)	8.2 ± 0.8	4.7 ± 0.01	3.0 ± 0.01 ^1^	4.7 ± 0.03	5.2 ± 0.04 ^2,3^
Carbohydrate (% of total energy)	42.5 ± 2.7	46.4 ± 0.6	61.7 ± 0.02 ^1^	46.9 ± 0.01	27.4 ± 0.05 ^2,3^
Protein (% of total energy)	18.9 ± 1.3	17.9 ± 0.01	18.1 ± 0.02 ^1^	17.8 ± 0.01	17.8 ± 0.03 ^3^
Cholesterol (mg/day)	479 ± 93	378 ± 10	492 ± 21 ^1^	352 ± 11	506 ± 17 ^2^
Total fiber (g/day)	16.2 ± 2.1	47.2 ± 2.0	46.1 ± 2.1	45.8 ± 1.8	39.8 ± 1.3 ^2,3^
Fructose (g/day)	26.7 ± 7.6	34.1 ± 1.8	46.1 ± 2.1 ^1^	33.1 ± 1.4	10.0 ± 0.3 ^2,3^
Vitamin C (mg/day)	68.8 ± 20.0	260.8 ± 13.0	306.6 ± 16.2 ^1^	230.4 ± 20.8	121.1 ± 4.8 ^2,3^
Vitamin E (mg/day)	9.0 ± 1.7	27.4 ± 1.1	14.4 ± 0.6 ^1^	24.8 ± 1.6	28.5 ± 0.8 ^2,3^

Data for the study diet composition is inclusive of all subjects who completed the control and corresponding LFD or HFD (*n* = 10 for each). Mean diet composition data for the subset of subjects (*n* = 7) who completed both diet protocols is not listed separately here, but was similar to those who completed only one of the intervention diets. All data are reported as mean ± SEM. ^a^ Food record data were only available on 11 subjects. Abbreviations: MUFA = mono-unsaturated fatty acids, PUFA = poly-unsaturated fatty acids. ^1^ *p* < 0.017 compared to CONT LFD.^2^ *p* < 0.017 compared to CONT HFD.^3^ *p* < 0.017 HFD *vs.* LFD for the 7 subjects who completed both diet interventions.

At baseline, subjects completed a 3-day food record. Fructose values were estimated from the USDA Standard Reference 18–20 (USDA Database for the Added Sugar Content of Selected Foods, Release 1, 2006) [9] and Food and Nutrient Database for Dietary Studies 2.0 [10]. Caloric needs were estimated using the average of the Mifflin-St. Jeor [11] and Harris-Benedict [12] equations, adjusted for physical activity. All food was prepared in the University of Washington Nutrition Research Kitchen. Subjects picked up their food and were weighed twice weekly, and caloric intake was adjusted to achieve weight stability. Subjects were instructed to maintain regular physical activity and to eat all of the food provided, not to eat any non-study food, and to report any deviations from the diet. Physical activity was assessed at the end of each control diet and each intervention diet by use of the short form seven-day international physical activity questionnaire (IPAQ) [13]. To determine compliance, subjects recorded all food consumed each day using a checklist which was returned to the nutritionist.

### 2.4. Study Procedures

Study procedures were performed at the end of the CONT diet and at the end of the LFD or HFD.

### 2.5. Quantification of Liver Fat

Magnetic resonance spectroscopy (MRS) was used to quantify hepatic triglyceride using a Philips Achieva 3 Tesla, version 2.5.3.0 (Philips Healthcare, Andover, MA, USA) whole body scanner as previously described [8]. A single radiologist blinded to the diet intervention interpreted the scans. Hepatic lipid content is expressed as percent fat by weight. The inter- and intra-scan coefficients of variation (CVs) for liver fat were 18.6% and 1.2% respectively.

### 2.6. Body Fat Distribution

Total fat and lean mass were determined on the first control diet by Dual Energy X-Ray Absorptiometry using the QDR^®^ 4500A bone densitometer system (Hologic, Inc., Bedford, MA, USA).

Abdominal fat distribution was measured using MRI abdominal images as previously described [8]. Intra-abdominal fat (IAF) and abdominal subcutaneous fat (SQF) volumes were calculated by a single radiologist blinded to diet assignment using software fslview V3.1 (FMRIB Analysis Group, Oxford, UK) [14] combined with custom software. The inter- and intra-scan CVs were 4.9% and 2.4% for IAF and 6.2% and 3.1% for SQF respectively.

### 2.7. Assays

The following assays were performed: glucose in triplicate by glucose oxidase with intra- and inter-assay CVs of <1.7% and 1.7%; insulin in duplicate by automated electrochemiluminescence immunoassay with intra- and inter-assay CVs of 2.1% and 3.9% (Cobas e601, Indianapolis, IN, USA); highly sensitive C-reactive protein (hsCRP) by nephelometry with intra- and inter-assay CVs of 2.7% and 3.0% (Siemens, Tarrytown, NY, USA); adiponectin in duplicate by radioimmunoassay with intra- and inter-assay CVs of 6.2% and 9.3% (Millipore, Billerica, MA, USA); samples for IL-6, IL-10, IL-12 and gamma-interferon were run in duplicate with each individual’s samples run together in the same assay by ELISA with intra-assay CVs of 2.2%, 2.6%, 3.7% and 3.1% respectively (eBioscience, San Diego, CA, USA); total and HDL cholesterol were analyzed in the clinical laboratory using an enzymatic colorimetric Roche Cobas c501 assay (F. Hoffmann-La Roche, Basel, Switzerland) with LDL cholesterol calculated using Friedewald’s formula; total nonesterified free fatty acids (NEFAs) using HR Series NEFA-HR kit (Wako Diagnostics Home, Richmond, VA, USA) with intra- and inter-assay CVs of 0.8% and 3.7%. Fasting urinary F2-α isoprostanes were measured by gas chromatography/mass spectroscopy (GC/MS) using the procedure detailed by D. Milatovic *et al.* [15]. GC/MS analysis was conducted using a 6890N Agilent gas chromatograph coupled to a 5973 quadrupole mass spectrometer (Agilent Technologies, Santa Clara, CA, USA) in the negative-ion mode. Areas under peaks for *m*/*z* 569.5 and 573.2 (internal standard) were manually integrated to quantify both analytes. Urinary isoprostanes concentrations were corrected for urine creatinine concentrations. 

### 2.8. Statistical Analysis

Statistical analyses were performed using SPSS software (V19.0, SPSS Inc., Chicago, IL, USA). Non-normal data were log_e_ transformed. Comparisons of diet composition between the study diets were performed using paired *t*-test analysis with a *p* value less than 0.017 considered significant to account for three comparisons. Generalized Estimating Equation (GEE) analysis was performed to determine the effect of diet type on the change in each outcome variable (intervention diet—respective control diet), adjusted for diet order. Bonferroni correction for multiple comparisons was applied. A *p* < 0.05 was considered significant.

## 3. Results

### 3.1. Baseline Subject Characteristics and Diet Composition

A total of 13 subjects (10M/3F: 3 African Americans, 1 Asian and 9 Caucasians; age 36 ± 2.9 years; BMI 33.6 ± 1.3 kg/m^2^; fasting glucose 90.3 ± 1.9 mg/dL; 2-h glucose 94.2 ± 6.0 mg/dL) completed study diets and procedures. NAFLD (liver fat >5%) was present in 7/13 subjects. All three African Americans had <5% liver fat.

Analysis of baseline food records and study diet content and composition is reported in Table 1. Protein, fiber and cholesterol content were fairly well matched across study diets. Servings of fruit could not be matched due to differences in carbohydrate content of the LFD and HFD. This resulted in little to no fruit on the HFD and more fruit on the LFD compared to the CONT diet. As a result, fructose content and vitamin C were higher on the LFD and lower on the HFD. However, the absolute amounts of fructose consumed were kept relatively low on both the LFD and HFD. MUFA and PUFA content were lower on the LFD and higher on the HFD due to overall differences in fat content resulting in differences in the fat-soluble antioxidant, vitamin E.

All participants except two reported consuming all food provided. One subject had a short illness while on the LFD diet. Her recorded diet intake revealed lower calorie intake, but macronutrient composition similar to the prescribed diet (18.9% protein, 18.8% fat and 62.3% carbohydrate). The second subject failed to pick up his food once during the LFD and thus was off diet for 2 days.

There was no significant change in physical activity between the control diet and the corresponding intervention diets (*p* > 0.2 for all comparisons, data not shown).

### 3.2. Response to the LFD

**Figure 1:**
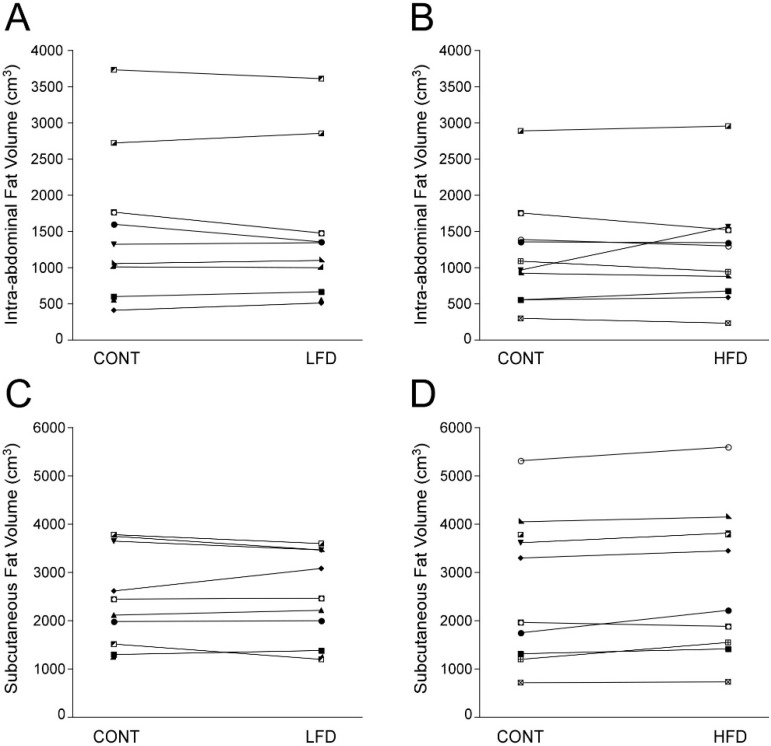
Effect of dietary fat on abdominal fat distribution. Compared to the control (CONT) diet, intra-abdominal fat (IAF) volume did not change on either diet (Panels **A** and **B**). Abdominal subcutaneous fat (SQF) increased on the high fat diet (HFD) (mean (95% CI): change 156 (73, 239) cm^3^), but did not change on the low fat diet (LFD) (panels **C** and **D**).

**Figure 2:**
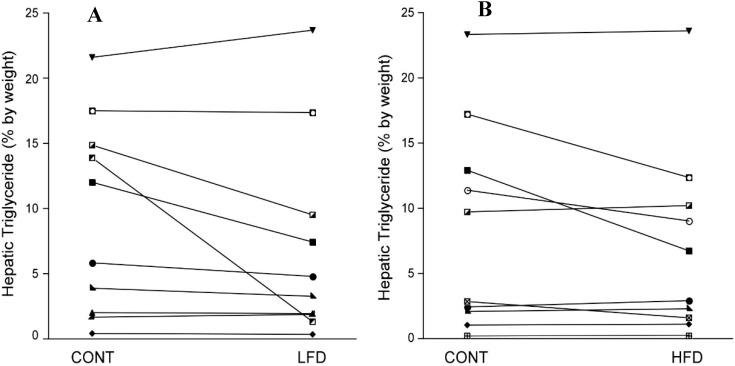
Effect of dietary fat on hepatic triglyceride. Compared to the CONT diet, hepatic triglyceride content by MRS decreased on the LFD (Panel **A**: mean (95% CI): change −2.13% (−3.74%, −0.52%)), but did not change on the HFD (Panel **B**). One subject on the LFD had a dramatic decrease in hepatic triglyceride from 13.9% to 1.3%. Removal of this subject decreased the mean change, but there was still a significant decrease from the CONT diet (Panel **A**: change −1.18% (−2.11%, −0.25%)).

Body weight, IAF and SQF remained stable during the LFD compared to CONT (Table 2; Figure 1). The LFD resulted in a statistically significant 13.9 ± 10.2% relative decrease in hepatic triglyceride content (mean (95% CI): absolute change −2.13% (−3.74%, −0.52%)) (Table 2; Figure 2), but no change in metabolic parameters, inflammatory markers or urinary F2-α isoprostanes.

### 3.3. Response to the HFD

Weight and IAF remained stable on the HFD, but SQF increased significantly compared to CONT (Table 2; Figure 1). There were no significant changes in hepatic triglyceride content (mean (95% CI): change −1.25% (−2.64%, 0.14%)) (Table 2; Figure 2), urinary F2-α isoprostane levels, metabolic variables, markers of inflammation or adipokines (Table 2).

**Table 2:** Effect of the diets on body weight, liver fat, abdominal fat distribution and metabolic parameters.

**Title**	**Low Fat Diet**		**High Fat Diet**	
	**CONT**	**LFD**	**CONT**	**HFD**
Weight (kg)	100.7 ± 4.1	100.5 ± 4.4	104.0 ± 5.8	104.1 ± 5.9
Liver fat (%)	9.4 ± 7.5	7.2 ± 7.7 ^1^	8.3 ± 7.9	7.0 ± 7.2
IAF (cm^3^)	1479 ± 331	1447 ± 321	1179 ± 235	1202 ± 238
SQF (cm^3^)	2440 ± 316	2413 ± 301	2704 ± 478	2861 ± 483 ^1,2^
ALT (Units/L)	26.2 ± 2.8	23.8 ± 2.9	25.2 ± 3.0	25.4 ± 2.1
AST (Units/L)	21 ± 1.6	19.0 ± 1.5	20.6 ± 1.9	22.2±2.7
GGT (Units/L)	30.8 ± 6.0	33.7 ± 10.2	29.0 ± 7.2	26.8 ± 8.0 ^2^
Total cholesterol (mg/dL)	179.1 ± 6.0	179.4 ± 7.3	159.3 ± 6.2	166.5 ± 8.8
Triglycerides (mg/dL)	120.1 ± 10.7	148.7 ± 25.4	84.2 ± 9.8	88.6 ± 14.1
HDL cholesterol (mg/dL)	41.5 ± 4.4	40.0 ± 4.6	48.1 ± 4.8	47.4 ± 5.3
LDL cholesterol (mg/dL)	113.6 ± 6.8	109.7 ± 6.9	94.4 ± 6.8	101.3 ± 8.6
Fasting glucose (mg/dL)	95.5 ± 3.3	97.1 ± 4.0	95.3 ± 3.3	94.7 ± 2.9
Fasting insulin (µU/mL)	13.9 ± 2.1	12.8 ± 2.0	16.6 ± 3.7	15.0 ± 3.3
Fasting NEFA (mE/L)	0.39 ± 0.04	0.39 ± 0.06	0.34 ± 0.04	0.35 ± 0.04
Adiponectin (µg/mL)	3.4 ± 0.3	4.1 ± 1.2	4.2 ± 0.9	4.6 ± 1.2
Leptin (ng/mL)	13.9 ± 3.3	15.1 ± 3.3	17.3 ± 3.5	16.8 ± 4.1
hsCRP (mg/L)	3.3 ± 0.9	2.8 ± 0.8	2.3 ± 0.6	2.2 ± 0.4
IL-6 (pg/mL)	1.08 (1.09)	1.01 (1.14)	0.91 (1.4)	0.83 (2.4)
IL-10 (pg/mL)	3.7 ± 0.3	3.4 ± 0.2	3.2 ± 0.1	3.4 ± 0.2
IL-12 (pg/mL)	1.24 (3.02)	1.14 (4.57)	1.12 (6.35)	1.04 (11.01)
γ-interferon (pg/mL)	11.1 (19.0)	14.2 (15.2)	13.7 (21.4)	9.9 (19.8)
Urinary F2α isoprostanes (ng/mg Cr)	1.04 ± 0.12	1.00 ± 0.11	1.3 ± 0.2	1.3 ± 0.2

Data are presented as Mean ± SEM or Median (interquartile range) for non-normally distributed data for all subjects who completed the control and corresponding LFD or HFD. Mean data for the seven subjects who completed both diets are not shown separately.^1^ *p* < 0.05 for significant change from respective CONT; ^2^ *p* < 0.05 LFD *vs.* HFD in the subset who completed both interventions (*n* = 7). Abbreviations: IAF = intra-abdominal fat, SQF = subcutaneous fat, ALT = alanine aminotransferase, AST = aspartate aminotransferase, GGT = gamma-glutamyl transferase, NEFA = non-esterified fatty acids, hsCRP = high sensitivity C-reactive protein, IL-6 = interleukin-6, IL-10 = interleukin-10, IL-12 = interleukin-12.

### 3.4. Comparison of the LFD and HFD

There was no main effect of diet type on the change in hepatic triglyceride content (*p* = 0.39) or IAF (*p* = 0.19), but there was a significant effect of diet type on SQF with the HFD resulting in an increase in SQF (*p* = 0.02). There was no effect of diet type on ALT or AST, but a trend for GGT (*p* = 0.05) favoring an increase in GGT on the LFD compared to the HFD. There was no significant effect of diet type on other metabolic variables, markers of inflammation, urinary isoprostanes or adipokines.

## 4. Discussion

We demonstrate that in weight stable individuals, a diet with a very high fat and saturated fat content did not increase hepatic triglyceride content. We observed an increase in SQF on the HFD suggesting appropriate storage of excess dietary fat, even in those with NAFLD. We confirmed our previous finding that a diet low in fat and saturated fat results in a modest decrease in hepatic triglyceride content. Surprisingly, neither diet intervention impacted markers of inflammation or oxidative stress, both key features of NASH.

Although the data shown in Table 1 suggest that total energy intake was substantially higher on the study diets compared to the baseline diet, it is important to emphasize that the baseline dietary intake data were estimates based on a 3-day dietary record; it is well known that this dietary assessment instrument underestimates energy intake, particularly in overweight and obese individuals [16]. In contrast, the energy intake data on the study diets were based on reported consumption of weighed foods provided by a nutrition research kitchen and therefore likely much more reliable. The fact that body weight did not change significantly suggests that we were able to administer the diets in isocaloric and energy balanced fashion.

The lack of an increase in hepatic triglyceride content on the HFD despite very high fat and saturated fat content is consistent with our previous findings using more modest fat content (45% calories from fat) [8]. These observations are contrary to previous studies that showed an increase in hepatic triglyceride content on a HFD compared to a LFD [6,7]. These previous studies used similarly high fat/saturated fat content and saw significant changes after only two or three weeks. Thus, the four weeks in our study should have been adequate time to observe a change in hepatic triglyceride content. The fact that hepatic triglyceride decreased on the LFD but did not change on the HFD suggests that either (a) the relationship between dietary fat/SFA intake and hepatic triglyceride is not linear and/or (b) factors other than total fat and saturated fat content of the HFD contribute to hepatic triglyceride content. Differences in fat and carbohydrate composition, age and underlying glucose metabolism status may have contributed to the discrepant findings between studies. For example, the higher MUFA content on our HFD might have had a protective effect as a high MUFA diet (27% MUFA/6.7% saturated fat) was shown to decrease liver fat in subjects with type 2 diabetes [17]. Similarly, our HFD and LFD were matched for dietary fiber content, resulting in an unusually high fiber intake in the HFD arm. It is possible that this higher fiber intake might have had a protective effect. We also cannot completely exclude a beneficial effect of lower fructose in the HFD that might have counterbalanced negative effects of the high fat content on liver fat. Whether a diet specifically designed to be low in fructose is able to decrease liver fat in humans is currently under investigation.

Our finding of a modest, 14% relative decrease in hepatic triglyceride on the LFD confirms similar findings in our previous study in which a 20% relative decrease compared to baseline diet was observed [8], but in a younger, more overweight/obese group with higher hepatic triglyceride content at baseline. We doubt that the higher fructose content in the LFD impacted our results as the average difference between the CONT and LFD diet was only 12 g per day and both were relatively low in fructose. Previous studies in humans used much higher amounts of fructose and only observed changes in liver fat under hypercaloric conditions. One week on a hypercaloric, high fructose diet (3 g fructose/kg/day [18] and 3.5 g fructose/kg fat free mass/day [19]) resulted in adverse effects on glucose metabolism, increased *de novo* lipogenesis [18] and increased intrahepatic lipids [19]. However, two weight-stable studies that provided extra fructose (1.5 g fructose/kg/day [20] and 150 g fructose/day [21]) for four weeks found no changes in liver fat.

Increased markers of oxidative stress are a consistent observation in subjects with NAFLD and NASH compared to control subjects [22,23,24,25] and such stress is thought to play a role in the progression to NASH [3,26]. Lipid peroxidation is increased in NAFLD [27], which can lead to increased production of reactive oxygen species. Beneficial effects of the anti-oxidant vitamin E on liver fat and hepatocyte injury [28] lend further support for a deleterious role of oxidative stress in the liver. The higher Vitamin E content on the HFD may have had a protective effect and may have counterbalanced other detrimental effects of the high saturated fat content resulting in no overall change in oxidative stress on the HFD. Previous studies have shown that weight loss achieved by diet and exercise decreases markers of oxidative stress and inflammation in diabetic men [29] and overweight youth [30]. Contrary to these studies, subjects in our study remained weight stable and we did not observe any changes in markers of inflammation or oxidative stress. Diets supplemented with extra virgin olive oil or nuts have also been shown to decrease markers of oxidative stress in subjects at high cardiovascular risk [31]. However, similar to what was observed here on the HFD, a low carbohydrate, high fat diet did not impact oxidative stress in overweight/obese women [32].

The strengths of our study include the controlled diet intervention, weight stability and the use of MRS to quantify hepatic triglyceride content. The small sample size is a limitation, which may have precluded our ability to detect significant effect of the diet interventions on other outcome variables. A further limitation is that it is not possible to extend these findings to other groups, such as individuals with type 2 diabetes who may have different underlying mechanisms for hepatic triglyceride accumulation. We specifically selected subjects with normal glucose tolerance and normal liver enzymes. Thus, our findings may reflect a relatively healthier overweight/obese population that may be able to adapt to changes in dietary fat intake. However, 50% of study subjects had NAFLD and should have responded to the diet interventions if the dietary fat-to-carbohydrate ratio was a major underlying mechanism. Finally, the HFD contained very high fat and saturated fat content that is not usually consumed in the general population. However, this study design strengthens the negative findings regarding the impact of high fat and saturated fat diets on inflammation and hepatic triglyceride.

## 5. Conclusions

Based on the absence of a significant change in hepatic triglyceride content after a very high fat/high saturated fat diet, we conclude that it is unlikely that excess dietary fat in the absence of caloric excess is a major driver in the development of NAFLD in otherwise healthy overweight/obese subjects. However, a diet very low in fat and saturated fat showed a modest decrease in hepatic triglyceride content and thus over a longer period of time may protect against development or progression of NAFLD.

## Supplementary Files

Supplementary File 1
