# Supplementary material for: Effects of Dietary Fat and Saturated Fat Content on Liver Fat and Markers of Oxidative Stress in Overweight/Obese Men and Women under Weight-Stable Conditions
Source: Nutrients. 2014 Oct 28;6(11):4678–90. doi: 10.3390/nu6114678 (PMC4245556; doi:10.3390/nu6114678)
Supplement: Supplementary File 1 [file nutrients-06-04678-s001.docx]

**Supplementary Information**

**Table S1.** Control diet menu.

|  | **Breakfast** | **Lunch** | **Dinner** | **Snack 1** | **Snack 2** |
| --- | --- | --- | --- | --- | --- |
| Day 1 | Bagel  Jam  Cream cheese  Apple | Three bean salad  Turkey sandwich  Lettuce/tomato  Light mayonnaise | Rice noodle stir-fry | Orange  Nonfat milk | Blueberry muffin  Butter |
| Day 2 | Toast Eggbeaters Butter Apple | Couscous salad Cucumber Nonfat milk | Mushroom risotto  Sugar snap peas  Orange | Blackberry crisp | Cream cheese dip  Celery  Carrots  Bread |
| Day 3 | Oatmeal  Nonfat milk  Toast  Peanut butter  Apple | Hummus  Pita  Tomato  Cucumber  Feta | Chicken fajitas  Corn tortilla  Salsa  Sour cream | Yoghurt  Almonds  Raisins | Blueberry muffin  Butter |

**Table S2.** HFD menu.

|  | **Breakfast** | **Lunch** | **Dinner** | **Snack 1** | **Snack 2** |
| --- | --- | --- | --- | --- | --- |
| Day 1 | Toast  Butter  Eggbeaters  String cheese | Couscous salad  Broccoli | Lasagna  Lettuce  Carrots  Balsamic vinaigrette | Sugar cookie | Cream cheese dip  Celery Almonds |
| Day 2 | Lowfat milk  Blueberry muffin  Butter | Three bean salad  Turkey sandwich | Chicken fajitas  Sour cream  Salsa  Cheddar cheese  Lettuce/tomato  Cranberry vinaigrette | Peanut butter Graham crackers | Mini quiche |
| Day 3 | French toast  Butter  Syrup | Chicken casserole  Carrots | Mushroom risotto  Sugar snap peas | Lettuce  Cucumber  Balsamic vinaigrette | Cream cheese dip  Celery Almonds |
| Day 4 | Granola  Pecans  Almonds  Lowfat milk | Turkey vegetable soup  Lettuce, tomatoes  Cranberry vinaigrette | Rice noodle stir fry  Broccoli  Butter | Saltine crackers  Cheddar cheese  Carrots | Mini quiche |

**Table S3.** LFD menu.

|  | **Breakfast** | **Lunch** | **Dinner** | **Snack 1** | **Snack 2** |
| --- | --- | --- | --- | --- | --- |
| Day 1 | Bagel  Cream cheese  Applesauce  Nonfat milk | Turkey pita  Lettuce/tomato  Light mayonnaise  String cheese  Carrots | Chicken fajita  Corn tortilla  Salsa  Sour cream | Yogurt  Dried cranberries  Mandarin oranges | Cream cheese dip  Celery  Carrots  Orange |
| Day 2 | French toast  Butter  Syrup  Nonfat milk  Orange | Hummus  Pita  Carrots/cucumber  Tomatoes | Rice noodle stir-fry  Grapes Roll | Yogurt  Dried cranberries  Vanilla wafers | Blueberry muffin |
| Day 3 | Oatmeal  Nonfat milk  Applesauce  Dried cranberries  Dried apricots  Bread | Three bean salad  Roll  Butter | Chicken casserole Sugar snap peas | Pineapple Vanilla wafers | Cream cheese dip  Celery carrots  Orange |
| Day 4 | Breakfast quiche  Banana  Orange juice | Turkey vegetable soup  Bread  Cucumber  String cheese  Peaches | Mushroom risotto Broccoli | Blackberry crisp | Blueberry muffin |

© 2014 by the authors; licensee MDPI, Basel, Switzerland. This article is an open access article distributed under the terms and conditions of the Creative Commons Attribution license (http://creativecommons.org/licenses/by/4.0/).
